# Language Network Function in Young Children Born Very Preterm

**DOI:** 10.3389/fnhum.2018.00512

**Published:** 2018-12-20

**Authors:** Eun Jung Choi, Marlee M. Vandewouw, Julia M. Young, Margot J. Taylor

**Affiliations:** ^1^Department of Diagnostic Imaging, The Hospital for Sick Children, Toronto, ON, Canada; ^2^Bloorview Research Institute, Holland Bloorview Kids Rehabilitation Hospital, Toronto, ON, Canada; ^3^Neurosciences & Mental Health, SickKids Research Institute, Toronto, ON, Canada; ^4^Department of Psychology, University of Toronto, Toronto, ON, Canada; ^5^Department of Medical Imaging, University of Toronto, Toronto, ON, Canada

**Keywords:** very preterm-born, resting-state functional connectivity, language network, Broca’s area, Wernicke’s area, children

## Abstract

Language deficits are reported in preterm born children across development. Recent neuroimaging studies have found functional alterations in large-scale brain networks underlying these language deficits, but the early childhood development of the language network has not been investigated. Here, we compared intrinsic language network connectivity in 4-year-old children born VPT and term-born controls, using defined language regions (Broca’s area, Wernicke’s areas, and their homologues in the right hemisphere). Resting-state functional magnetic resonance imaging (fMRI) was obtained, and the group differences in whole-brain connectivity were examined from each seed as well as correlations with language outcomes. We found significantly decreased functional connectivity in almost all language regions in children born VPT compared to their term controls. Notably, Broca’s area homologue in the right hemisphere emerged as a functional hub of decreased connectivity in VPT group, specifically to bilateral inferior frontal and supramarginal gyri; connectivity strength between Broca’s area homologue with the right supramarginal and the left inferior frontal gyri was associated with better language outcomes at 4 years of age. Wernicke’s area and its homologue also showed decreased inter-hemispheric connections to bilateral supramarginal gyri in the VPT group. Decreased intra- and inter-hemispheric connectivity among primary language regions suggests immature and altered function in the language network in children born VPT.

## Introduction

Every year, an estimated 15 million babies are born preterm and this number is rising (World Health Organization [WHO], 2018). At birth, preterm neonates suffer both major (8%) and minor (37%) medical morbidities (Manuck et al., 2016), but significant neurocognitive deficits are reported in childhood even among children who show no obvious neurological injury (Isaacs et al., 2004; Aarnoudse-Moens et al., 2012). In the language domain, preterm birth has been found to increase the risk of deficits in the preschool and school years (Sansavini et al., 2010; Gonzalez-Gomez and Nazzi, 2012). Poor language outcomes emerge as early as the first 2 years of life and are observed across development (Vohr, 2014). Some studies reported that language problems in preterm-born children may improve with age (Luu et al., 2009; Putnick et al., 2017). Others suggested, however, these early impairments in language do not improve, even though preterm infants experience an earlier and richer exposure to language (Peña et al., 2010, 2012), suggesting significant neurobiological changes in the brains of children born preterm. The risk for language difficulties increases with lower gestational age such that children born very preterm (VPT, <32 weeks of gestational age) experience significantly lower acquisition of vocabulary at 2 years of age, and lower scores in receptive and expressive language skills and phonological perception at school age (Foster-Cohen et al., 2007; Sansavini et al., 2010; Barre et al., 2011). In addition, a meta-analysis found that these language deficits increased between 3 and 12 years of age (van Noort-van der Spek et al., 2012). However, neurobiological mechanisms underlying these difficulties are still unknown.

Structural and functional alterations in the VPT brain have been reported as the underlying neural mechanisms that mediate neurocognitive deficits. Structural alterations may be due to disturbances in the considerable neural development, including myelination, proliferation, and synaptic organization, that occurs in the last trimester of pregnancy (Counsell et al., 2003), the period of very preterm birth. Individuals born VPT have shown reduced volumes in cortical, subcortical gray, and white matter as well as the cerebellum, compared to term-born controls (Parker et al., 2008; Carreiras et al., 2009; Ment et al., 2009; Young et al., 2015, 2018). Of note, results of volumetric and microstructural connectivity changes in language areas, including the left frontal regions, temporal, and parietal cortices, have been implicated in poor language function in this population (Lind et al., 2011; Nosarti et al., 2011).

Recent advances in functional neuroimaging techniques have highlighted resting-state functional connectivity, which measures the temporal correlations of blood-oxygen-level-dependent (BOLD) signal among spatially distributed brain regions; regions with correlated activity are believed to form intrinsic functional networks (Friston et al., 1996). This approach towards a systematic understanding of large-scale functional brain organization has had huge benefits in expanding our knowledge of neural mechanisms of various clinical conditions. A recent study, which reviewed atypical functional connectivity in individuals born preterm across development, suggested long-lasting changes in language organization from the fetal period to young adulthood (Kwon et al., 2016; Thomason et al., 2017). They showed that preterm infants demonstrated reduced connectivity in the language network compared to their term-born controls; less complexity at 38 weeks of gestational age (Smyser et al., 2014), decreased lateralization in left frontal-temporal language area, and no significant connectivity between left and right Broca’s area at 42 weeks of age (Kwon et al., 2015). However, other studies have found that older children and adolescents had evidence of both increased (Gozzo et al., 2009) and decreased connectivity in the language network (Scheinost et al., 2014). The functional alterations of language-related brain areas were also reported in young adulthood (Scheinost et al., 2012; Constable et al., 2013; Bäuml et al., 2014). Although converging evidence indicates an altered functional language network in individuals born preterm, we still lack knowledge on the effect of preterm birth on the language network development throughout the lifespan, with inconsistent results as well as significant gaps in early childhood.

Language ability develops very quickly during childhood (Berk, 2013), but the underlying neural bases are just starting to be investigated at a large-scale brain network level in young children. A recent study explored the developmental changes in intrinsic language network development in typically developing preschool children (Xiao et al., 2016a). They found that children at age 3 showed relatively strong contralateral connections in primary language regions compared to children at age 5. In addition, the developmental changes were characterized by increasing long-range connections between left inferior frontal gyrus and superior temporal gyrus and an increase in leftward lateralization. This result corresponds to typical language network maturation from inter- to intra-hemispheric connectivity (Friederici et al., 2011), with top-down processing centered in the inferior frontal cortex starting at this age (Skeide and Friederici, 2016), and an increase of leftward lateralization in Broca’s area (Tomasi and Volkow, 2012; Zhu et al., 2014). Given these behavioral and neural changes, children born preterm may have a different language network in early childhood compared to age-matched term-born children, contributing to their language difficulties; however, their intrinsic language network has not yet been examined.

We investigated the intrinsic language network across defined regions in children born VPT at 4 years of age compared to term-born controls, using resting-state functional magnetic resonance imaging (fMRI). We hypothesized altered connectivity in this network in children born VPT, specifically in primary language regions: Broca’s area, Wernicke’s area, and their homologues in the right hemisphere. Given the developmental pattern of maturation in older VPT children, we expected children born VPT would show decreased interhemispheric connectivity across the language regions compared to term-born children, and that the altered functional connectivity in VPT group may be associated with poorer language outcomes.

## Materials and Methods

### Participants

Forty-one children born very preterm [VPT: less than 32 weeks of gestational age (GA) (mean 28 weeks of GA)] participated in this study at 4 years of age (chronological age mean 4.2 ± 0.2 years) (Table 1). These children were part of a longitudinal investigation of children born VPT, followed every 2 years from birth. Thirty-two full-term (FT) born children were also recruited as controls (age mean 4.5 ± 0.4 years), through advertisements placed in the community, local schools, and at the hospital. Although all of the children in both groups were between 4 and 5 years of age, the term-born children were older by a few months than children born very preterm (*t* = -3.393, *p* < 0.01), so age was included as a covariate in all subsequent analyses. Exclusion criteria for both groups included a history of neurological or neurodevelopmental disorders, an IQ ≤ 70, and any language or vision issues preventing successful completion of tasks as well as standard MRI exclusions. All children gave verbal assent and parents gave written informed consent. This study was approved by the Research Ethics Board at the Hospital for Sick Children.

**Table 1:** Demographics and head motions of children born VPT and FT in final analysis.

		VPT	FT
Sample size	*n*	31	23
Age (years)	Mean ± SD	4.2 ± 0.2	4.5 ± 0.4
Sex	M:F	12:19	13:10
Gestational age (weeks)	Mean ± SD	28.3 ± 1.7	40 ± 1.5
Excluded volumes (out of 120)	Mean ± SD	1.61 ± 4.51	1.52 ± 2.71
Mean maximum displacement in head motions	Mean ± SD	0.25 ± 0.31	0.29 ± 0.20


### Neuropsychological Assessments

A measure of IQ was obtained for all 54 children using the Wechsler Preschool and Primary Scales of Intelligence – Third Edition (WPPSI-III; Wechsler, 2002), which included the Verbal (VIQ), Performance (PIQ), Processing Speed (PSQ), and Full-Scale IQ (FSIQ). Children’s language outcomes were assessed by Clinical Evaluation of Language Fundamentals (CELF Pre-2; Wiig et al., 2004), which included a Core Language score (CL), Receptive Language index (RL), Expressive Language index (EL), Language Content Index (LC), and Language Structure index (LS). Visual motor integration was also assessed using the Beery-Buktenica Test of Visual Motor Integration (VMI) (Beery et al., 2004).

### Data Acquisition

MRI data were acquired on a Siemens 3T Trio. A high resolution T1-weighted anatomical image was acquired [TR/TE = 2300/2.96 ms; FA = 9°; FOV = 192 mm × 240 mm × 256 mm; 1 mm isotropic voxels; 5 min acquisition time]. Resting state functional images were obtained with a blood oxygenation level dependent (BOLD) contrast gradient echo planar imaging pulse sequence [TR/TE = 2340/30 ms; FA 70°; FOV, matrix size = 64 × 64 × 40; 3.5 mm isotropic voxels; 120 volumes; 5 min acquisition time]. The children watched a movie of their choice during the structural images through MRI-compatible goggles, and the movie was replaced with a fixation cross inside a circle centred on the screen for the resting state scan.

### Data Preprocessing

All fMRI data were preprocessed in a locally developed pipeline using standard AFNI^1^ and FSL^2^ tools. The first four volumes at the beginning of the time series were dropped as scanner stabilization, leaving 116 volumes for analysis. Images were slice-time and motion corrected. We estimated spatial deviations between the reference functional image and other images using each of six motion parameters. The deviation for each image was calculated and output into a motion file, which then was used to censor timepoints that contained too much motion. Volumes in excess of 2 mm from the maximum mean displacement were discarded, and children who had more than 1/3 of their volumes removed were excluded from the analysis. The original sample included 73 children (VPT = 41, FT = 32), but 19 were excluded due to motion, leaving the final sample of 54 (VPT = 31, FT = 23) for analyses. When we examined the number of excluded volumes and the mean displacement of volumes for the remaining participants, there were no significant group differences in the number of excluded volumes and the mean maximum displacement (Table 1). The data were spatially smoothed using a 7-mm FWHM Gaussian kernel and band-pass temporal filtered (0.01–0.2 Hz) to remove magnetic field drifts of the scanner and minimize the physiological noise of high frequency components. Nuisance signals from white matter, cerebrospinal fluid, and head motion were regressed from the data.

### Data Analysis

We used a standard seed-voxel correlation approach to compare the functional connectivity of the language network between the two groups of children. Two cortical voxels that represent the primary language regions and their homologues in the right hemisphere were selected and used to create each subject’s ROIs (taken from Tomasi and Volkow, 2012); a voxel near the center of mass of the left pars triangularis (BA45; -51, 27, 18 mm; MNI coordinates), was selected to represent Broca’s area (Broca L), and its homologue in the right hemisphere (51, 27, 18 mm; MNI coordinates; Broca R). A voxel, in the left supramarginal gyrus (at the boundaries of BA39, BA4040, and BA20; -51, -51, 30 mm; MNI coordinates), was selected to represent Wernicke’s area (Wernicke L), and its homologue in the right hemisphere (51, -51, 30 mm; MNI coordinates; Wernicke R). To account for inter-subject variability, the seed coordinates were dilated within a 10-mm spherical ROI until the internal correlation dropped below 0.7 or the number of voxels exceeded 300 (see Supplement 1 for more details). First-level analysis was performed using FSL’s FEAT^3^ by correlating the mean time series of the ROIs with the time series of all voxels in the brain in each child. The first-level language network connectivity maps between preterms and controls were contrasted with covariates of age and sex, and the group mean mixed effects Z (Gaussianized T/F statistics; Jenkinson and Woolrich, 2000) statistic images were thresholded using clusters determined by *Z* > 3.29 and a corrected cluster significance threshold of FWE *p* < 0.05 assuming a Gaussian random field for the *Z*-statistics (Worsley et al., 1992). In addition, children’s neuropsychological characteristics, such verbal IQ (VIQ) scores, language outcomes (CL, RL, EL, LC, and LS), and gestational age were also explored in correlation with each subject’s language network connectivity.

## Results

### Behavioral Measures

Children born VPT showed significantly lower scores than FT controls in the PIQ (*t* = -3.975, *p* < 0.001), PSQ (*t* = -3.656, *p* < 0.001), and FSIQ (*t* = -3.779, *p* < 0.001) (Table 2). For VIQ, however, there was only a trend toward a difference between the two groups and seemed partly attributable to the wide score range, from 66 to 133, in children born VPT (*t* = -1.919, *p* < 0.10). Children born VPT also scored significantly lower in all sub-scores of the CELF, as well as on the VMI (see Table 2).

**Table 2:** Neuropsychological assessments.

	VPT (Mean ± SD)	FT (Mean ± SD)	*p*
**Wechsler preschool and primary scales of intelligence - third edition (WPPSI-III)**			
Verbal IQ (VIQ)	98.8 ± 18.1	106.7 ± 11.6	*t* = -1.919+
Performance (PIQ)	94.6 ± 11.7	107.5 ± 11.7	*t* = -3.975^∗∗∗^
Processing speed (PSQ)	92.2 ± 18.7	108.8 ± 9.9	*t* = -3.656^∗∗∗^
Full-scale (FSIQ)	94.0 ± 14.2	108.2 ± 12.4	*t* = -3.779^∗∗∗^
**(CELF Pre-2)**			
Core language score (CL)	95.31 ± 16.10	109.59 ± 12.13	*t* = -3.120^∗∗^
Receptive Language Index (RL)	96.0 ± 18.2	116.6 ± 7.7	*t* = -4.547^∗∗∗^
Expressive Language Index (EL)	98.5 ± 17.2	115.3 ± 10.5	*t* = -2.849^∗∗^
Language contents Index (LC)	98.4 ± 16.7	114.9 ± 8.2	*t* = -3.791^∗∗∗^
Language Structure Index (LS)	96.0 ± 19.2	117.5 ± 10.9	*t* = -3.294^∗∗^
**Beery-Buktenica Developmental Test**			
Visual-motor integration (VMI)	99.10 ± 11.54	110.48 ± 8.93	*t* = -3.788^∗∗∗^


*^∗∗∗^p < 0.001, ^∗∗^p < 0.01, +p < 0.10.*

### Group Differences in Primary Language Networks

VPT children showed decreased functional connectivity overall in the primary language regions compared to FT controls (The group mean connectivity for each seed is presented in Supplement 2). Notably, connectivity from Broca’s region (Broca L) did not show significant group differences, while the connectivity from other ROIs (Broca R, Wernicke L, and Wernicke R) was decreased in children born VPT compared with FT controls.

**FIGURE 1:**
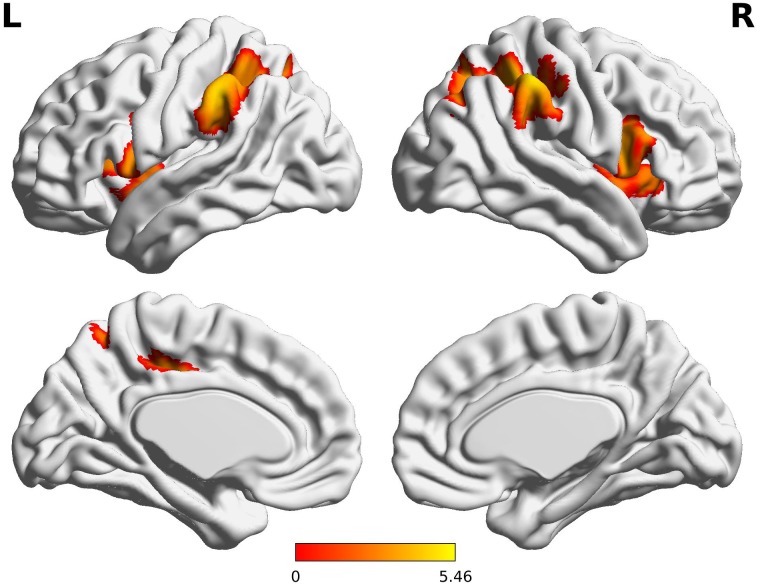
Decreased functional connectivity from Broca’s area homologue in children born VPT. Red-yellow regions show group differences of significantly decreased functional connectivity from Broca’s area homologue in children born VPT.

Compared to the FT group, VPT children showed significant reductions in connectivity between the Broca R to inferior frontal gyri (BA44), insulae (BA13), supramarginal gyri (BA40), and superior parietal lobules (BA7) bilaterally, as well as to the left posterior cingulate cortex (BA31) (*Z* > 3.29, FWE *p* < 0.05; Table 3 and Figure 1).

**Table 3:** Peak voxels with decreased functional connectivity from Broca’s area homologue in children born VPT compared to full-term children.

Cluster	Peaks	L/R	BA	MNI coordinates			*Z*
				*x*	*y*	*z*	
1	Supramarginal gyrus	L	40	-60	-34	42	4.68
		L	40	-44	-46	54	3.67
		L	40	-32	-36	40	3.33
	Posterior cingulate cortex	L	31	-14	-26	40	3.64
2	Supramarginal gyrus	R	40	44	-40	46	4.24
	Inferior parietal lobule	R	7	22	-64	44	3.67
		R	7	36	-72	36	3.4
3	Inferior frontal gyrus	R	44	48	14	12	4.42
		R	44	44	16	0	3.16
	Insula	R	13	44	4	8	3.21
4	Inferior frontal gyrus	L	44	-48	4	8	3.52
			44	-44	2	26	3.37
	Insula	L	13	-42	10	-14	3.42


*L/R, left hemisphere/right hemisphere; BA, Brodmann area; Z > 3.29, FWE, p < 0.05.*

**FIGURE 2:**
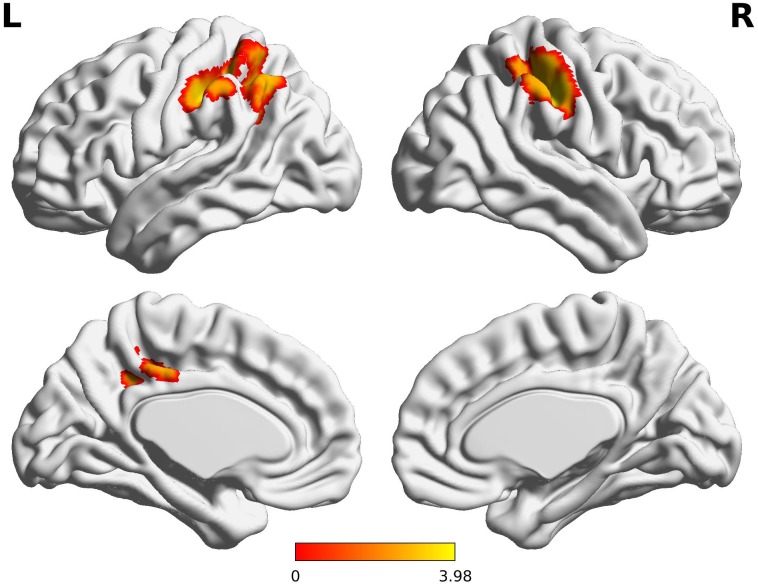
Decreased functional connectivity from Wernicke’s area in children born VPT. Red-yellow regions show group differences of significantly decreased functional connectivity from Wernicke’s area in children born VPT.

The Wernicke L connectivity to bilateral supramarginal gyri (BA40) and superior parietal lobule (BA7) was decreased in children born VPT, as well as the left angular gyrus (BA39) and the left posterior cingulate cortex (BA31) and the right postcentral gyrus (BA1) (*Z* > 3.29, FWE *p* < 0.05; Table 4 and Figure 2).

**Table 4:** Decreased functional connectivity from Wernicke’s area in children born VPT, compared to full-term children.

Cluster	Peaks	L/R	BA	MNI coordinates			*Z*
				*x*	*y*	*z*	
1	Supramarginal gyrus	L	40	-46	-26	38	3.68
		L	40	-50	-24	40	3.63
		L	40	-34	-36	44	3.41
	Angular gyrus	L	39	-50	-54	46	3.28
2	Postcentral gyrus	R	1	48	-24	40	3.98
		R	1	60	-20	44	3.61
		R	1	54	-26	54	3.43
		R	1	38	-30	46	3.41
	Supramarginal gyrus	R	40	58	-36	58	3.75
		R	40	58	-28	56	3.48


*L/R, left hemisphere/right hemisphere; BA, Brodmann area; Z > 3.29, FWE p < 0.05.*

**FIGURE 3:**
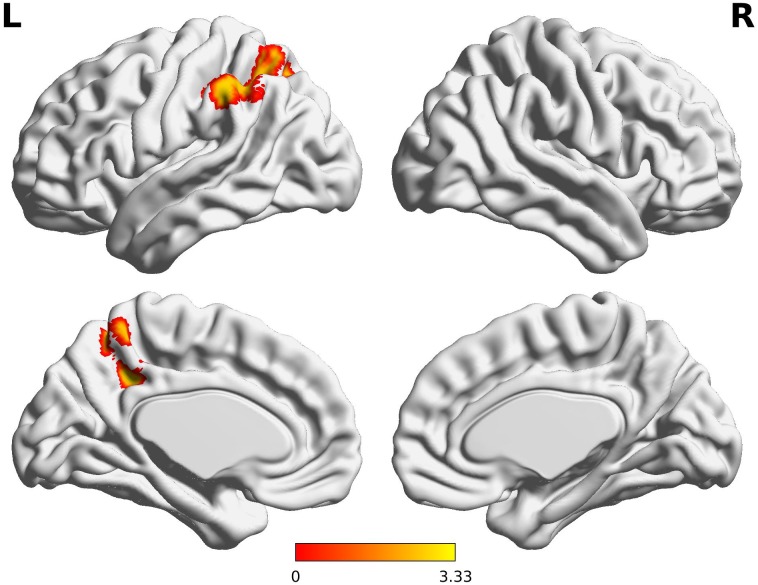
Decreased functional connectivity from Wernicke’s area homologue in children born VPT. Red-yellow regions show group differences of significantly decreased functional connectivity from Wernicke’s area homologue in children born VPT.

The decreased pattern of Wernicke R connectivity was seen only in contralateral connections in the VPT children, which were to the left supramarginal gyrus (BA40), left superior parietal lobule (BA7), posterior cingulate cortex (BA31), and precuneus (BA7) (*Z* > 3.29, FWE *p* < 0.05; Table 5 and Figure 3).

**Table 5:** Decreased functional connectivity from Wernicke’s area homologue in children born VPT, compared to full-term children.

Cluster	Peaks	L/R	BA	MNI coordinates			*Z*
				*x*	*y*	*z*	
1	Posterior cingulate cortex	L	31	-14	-44	38	3.33
	Inferior parietal lobule	L	1	-34	-32	40	3.07


*L/R, left hemisphere/right hemisphere; BA, Brodmann area; Z > 3.29, FWE p < 0.05.*

### Functional Connectivity Differences and Relations With Language Outcomes

There were significant correlations between some behavioral outcomes and the language network that showed significant group differences. In all children, only the core language scores were positively correlated with Broca R connectivity, with areas including the right SMG cluster (*r* = 0.380, *p* = 0.012), the left IFG cluster (*r* = 0.320, *p* = 0.037), and the right IFG cluster (*r* = 0.315, *p* = 0.040). In contrast, subjects’ verbal IQ scores demonstrated no significant correlations with any connectivity values which showed group differences. The visual-motor integration scores were positively correlated with Broca R connectivity strength with the left SMG cluster (*r* = 0.386, *p* = 0.005) and the right IFG cluster (*r* = 0.357, *p* = 0.010).

We also examined if behavioral measures correlated with primary language networks at the whole brain level. The connectivity between primary language regions and motor/visual regions were only correlated with core language scores (CL) for both groups. The Broca L connectivity with premotor cortex (BA6) and visual cortices (BA17 /BA19) were significantly correlated with CL. The Broca R connectivity with premotor cortex (BA6) and postcentral gyrus (BA1) was also correlated with CL. In addition, Wernicke L but not R connectivity with visual area (BA18) was correlated with CL. When we examined the correlations between each language network and children’s VIQ and GA, no significant correlations were found (see Supplement 3, 4 for more details).

## Discussion

The present study investigated language network connectivity in 4-year-old children born VPT and term-born controls born at term using defined language regions (Broca’s and Wernicke’s areas) and their homologues in the right hemisphere. We found significantly decreased functional connectivity in language networks in children born VPT compared to their term born peers, extending the previous literature in older children, which showed atypical functional connectivity in intrinsic language networks with decreased or increased patterns (Gozzo et al., 2009; Myers et al., 2010; Kwon et al., 2015), to this pre-school age range. A decrease in voxel-wise connectivity may reflect subtle injury or immaturity in regions of the brain resulting in vulnerability in the establishment of long-range connections (Kwon et al., 2015). Over time, injured or immature brain areas may also show increased patterns of connectivity, as the typically developing brain reduces connections between proximal regions or contralateral homologues to strengthen connections between remote regions by the pruning of the initial overabundance of synapses (Kesler et al., 2004; Ment and Vohr, 2008). Given these developmental trajectories, our findings support the model of atypical functional organization of intrinsic language network in children born very preterm in early childhood.

In our study, Broca’s area homologue in the right hemisphere showed decreased connectivity to most primary language regions in children born VPT, with reduced connectivity to bilateral inferior frontal gyri (IFG) and bilateral supramarginal gyri (SMG). The atypical engagement of right-sided connectivity in the language network has been reported in children, adolescents, and young adults born preterm, suggesting that preterm birth affects developmental functional lateralization in the brain (Gozzo et al., 2009; Myers et al., 2010; Friederici et al., 2011; Scheinost et al., 2014). Given that the left–right asymmetry of the developing brain is mediated by programmed gene expression during the second and third trimester of gestation (Sun et al., 2005), preterm birth may alter this neurogenesis (Vaccarino and Ment, 2004; Malik et al., 2013) and result in long-lasting changes in functional organization. It is not clear, however, whether this pattern of connectivity represents a delay in cerebral maturation or a compensatory mechanism to overcome difficulties in language processing (Shaywitz et al., 2002; Rushe et al., 2004; Chilosi et al., 2005; Gozzo et al., 2009). Decreased connectivity from Broca’s right homologue in our study may reflect functional immaturities of the very preterm children at an early stage of language development, as cerebral language lateralization, dominant in left inferior frontal cortex and supporting top-down processing of language, is not yet fully specialized at this age (Skeide and Friederici, 2016). A compensatory strategy of right-sided language network in the preterm population could emerge gradually coupled with leftward lateralization and the development of top-down processing in later development.

Broca’s homologue in the right hemisphere showed significantly decreased inter-hemispheric and intra-hemispheric connections with bilateral IFG and SMG in the VPT group. There is evidence of strong inter-hemispheric functional connectivity between left and right IFG in typically developing children aged 3 to 5 years with a weakening pattern with increasing age (Xiao et al., 2016a). Six-year-old children still showed stronger inter-hemispheric connectivity compared to adults (Friederici et al., 2011). In children aged 5–6 years, the strong inter-hemispheric connection was correlated with more advanced language abilities (Xiao et al., 2016b). This strong inter-hemispheric correlation in typical development of the language network is partly supported by structural maturation of the splenium, through which the auditory commissures project and the interplay of left and right hemispheric language functions is possible (Dubois et al., 2008; Lebel et al., 2008). Thus, our findings of decreased inter-hemispheric connections from Broca’s homologue to left IFG indicates that preterm children are deviating from typical development at an early stage of life, and may be related to other findings of reductions in structural connectivity measures (Young et al., 2018). In addition, the decreased intra-hemispheric connectivity from Broca’s homologue to the right IFG and SMG may be further evidence of atypical language networks in children born VPT.

Typically developing children demonstrate a more right-sided functional lateralization during language processing compared to adults (Brauer and Friederici, 2007), and this may be related to children’s higher reliance on prosodic information during language processing, supported by the right hemisphere (Wartenburger et al., 2007; Sabisch et al., 2009). Even in the mature brain, suprasegmental prosodic information tends to be processed in the right superior temporal gyrus and the right inferior frontal gyrus. The right-dominant activation for phonological processing is also reported in young children (Sugiura et al., 2011). Given behavioral evidence that children born prematurely are at higher risk for delayed or atypical processing of phonetic and prosodic information (Gonzalez-Gomez and Nazzi, 2012; Ragó et al., 2014), decreased intra-hemispheric connection in the right hemisphere in children born VPT may underlie these specific deficits in their language development.

Both Wernicke’s area and its right hemisphere homologue also showed decreased inter-hemispheric connectivity to bilateral SMG in the VPT group, which represented alterations in shorter-range connectivity, compared to Broca’s area homologues which showed alterations in long range connectivity to bilateral SMG. In addition, Wernicke’s area also showed decreased intra-hemispheric connectivity to the left SMG and angular gyrus (AG). The bottom-up processes for cortical language networks, which involve low-level computation of mental representations from the sensory input, primarily implemented bilaterally in the temporal cortices, develop rapidly in first 3 years of life (Skeide and Friederici, 2016), and strong inter-hemispheric connections in these areas are observed from birth to early childhood. By 3 years of age, in typically developing children the intrinsic language network from the left posterior superior temporal gyrus is functionally correlated with bilateral inferior parietal lobe, left superior parietal lobe, and angular gyrus (Xiao et al., 2016a). At 6 years of age, children’s language network is still characterized by a strong functional inter-hemispheric connectivity, mainly between the superior temporal regions, compared to adults (Friederici et al., 2011). Given this typical developmental trajectory, preterm birth appears to disturb the development of bottom-up language processing network; these difficulties extend to older children and adolescents with reports of atypical connectivity between Wernicke’s area and SMG (Gozzo et al., 2009; Myers et al., 2010; van Ettinger-Veenstra et al., 2017).

Notably, the decreased connectivity from primary language regions in children born VPT consistently included the left SMG, which is widely accepted as a part of Wernicke’s area (Binder, 2015) and has traditionally been thought to be responsible for phonologic processing (Ment et al., 2006). This is also concordant with findings that reported atypical involvement of the left SMG with Broca’s area homologue in preterm children at school-age (Gozzo et al., 2009). Taken together, our results support the model that children born VPT have significant decreases in neural networks that support auditory phonologic processing of language.

The connectivity strength of Broca’s area homologue with primary language regions was positively correlated with children’s language outcomes. The better the language outcomes at 4 years of age, the higher the connectivity strength between Broca’s area homologue and the right SMG as well as between Broca’s area homologue and the left IFG. Interestingly, the primary language network connectivity at 4 years of age was associated with the core language score and the visual-motor integration score, which may reflect that the language network organization in the early childhood is related to general language ability as well as the coordination with sensory information processing and motor skills. The evidence of decreased connectivity in Broca’s area homologue in children born VPT in our study also points to the critical delays happening in this population.

There are several limitations to our study. A relatively small sample size may have restricted the generalizability of our findings to understanding children born very preterm. We included age as a covariate in our analyses since the term-born children were older than children born very preterm by a few months. We expect exactly age-matched controls in future studies would provide higher statistical power. Also most of our VPT sample showed only mild cognitive delays compared to their term-born peers. Future studies would require larger samples of children, and with a wider range of outcomes. Lastly, 5 min for rs-fMRI scans was a long time for our participants, as they were a very young clinical population, but we acknowledge that more volumes would be an advantage for analyses and stronger statistical power.

## Conclusion

In conclusion, we found atypical functional organization underlying language deficits in 4-year-old children born VPT, who showed decreased intra- and inter-hemispheric connectivity among primary language regions compared to their term-born peers. Broca’s area homologue in the right hemisphere showed the most marked functional connectivity effects, suggesting that preterm birth may cause fundamental alterations in the cerebral lateralization for language. Our findings demonstrate that altered functional connectivity in the language network in children born VPT is already present in the preschool age period and may represent neural bases of consistent lower language outcomes in this population.

## Author Contributions

MT and JY contributed to designing the study and collecting the data. EC and MV analyzed the data. EC, MV, and MT interpreted the results. EC wrote a draft of the manuscript. MV, JY, and MT reviewed and revised the manuscript. All authors confirmed the final version of the manuscript.

## Conflict of Interest Statement

The authors declare that the research was conducted in the absence of any commercial or financial relationships that could be construed as a potential conflict of interest.

## Footnotes

1 https://afni.nimh.nih.gov

2 https://www.fmrib.ox.ac.uk/fsl

3 https://fsl.fmrib.ox.ac.uk/fsl/fslwiki/FEAT
