# Supplementary material for: Language Network Function in Young Children Born Very Preterm
Source: Front Hum Neurosci. 2018 Dec 20;12:512. doi: 10.3389/fnhum.2018.00512 (PMC6306484; doi:10.3389/fnhum.2018.00512)
Supplement: Supplementary file 1 [file Data_Sheet_1.pdf]

Supplement 1. Location of seeds and ROIs

Seeds	Regions	xyz	BA
Broca's area	Left pars triangularis	-51, 27, 18	45
Broca's area homologue	Right pars triangularis	51, 27, 18	
Wernicke's area	Left supramarginal gyrus	-51, -51, 30	39,40,20
Wernicke's area homologue	Right supramarginal gyrus	51, -51, 30	

Supplement 2. Group mean connectivity in each language seed

2A. Group mean connectivity of
Broca' area seed

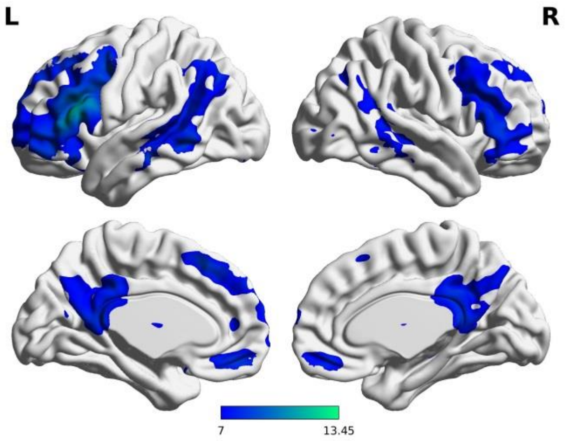

2B. Group mean connectivity of
Broca's area homologue seed

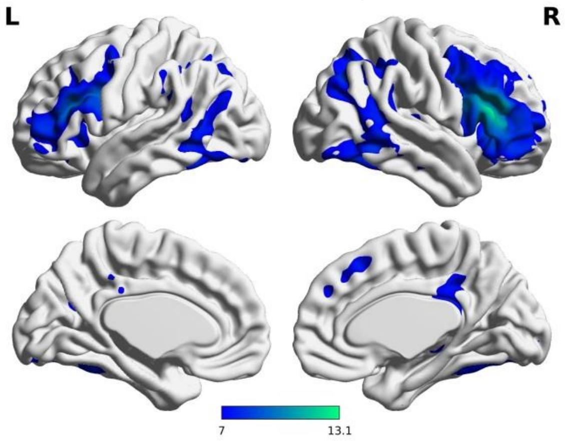

2C. Group mean connectivity of
Wernicke's area seed

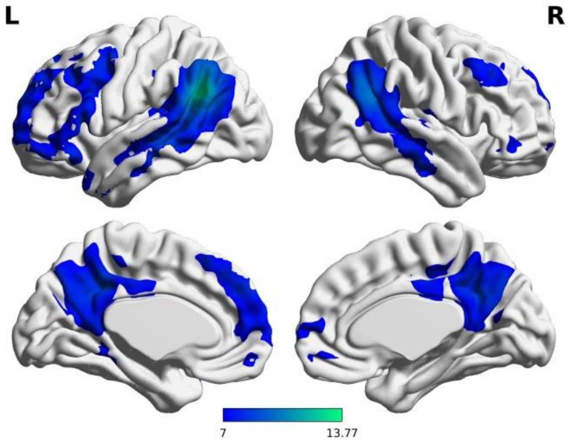

2D. Group mean connectivity of
Wernicke's area homologue seed

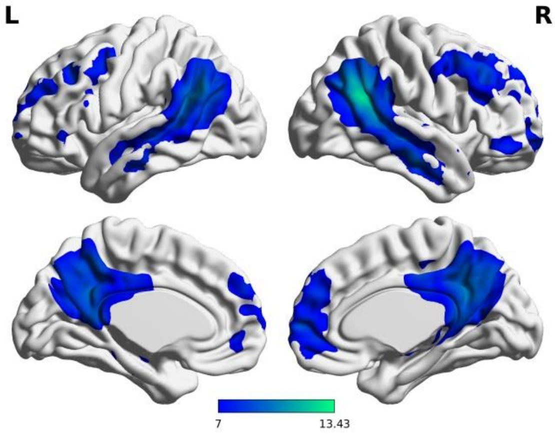

Blue regions represent the group mean connectivity for each seed across all children and each seed and ROIs are shown in green (Z>7.0, FWE p<.05). The Broca's area and its homologue in the right hemisphere demonstrated intra-hemispheric connectivity with the temporal and inferior parietal cortices. The Wernicke's area and its homologue in the right hemisphere demonstrated inter-hemispheric connectivity each other at 4 years of age.

Supplement 3. Peak voxels which showed correlations with language outcomes in the language networks

Cluster	peaks	L/R	BA	MNI coordinates			Z
				x	y	z	
Broca’s area connectivity							
1	Supplementary motor area	L	6	-18	-14	66	3.88
2	Lingual gyrus	L	19	-18	-52	-4	3.49
Broca’s area homologue connectivity							
1	Premotor cortex	L	6	-12	-4	62	3.25

L/R, left hemisphere/right hemisphere; BA, Brodmann area; Z>3.29, FWE p<.05

Supplement 4. Brain regions which showed correlations with language outcomes in the language network

4A. Broca’s area connectivity

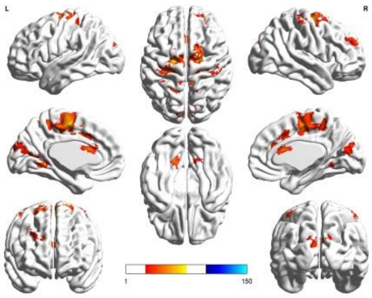

4B. Broca’s area homologue connectivity

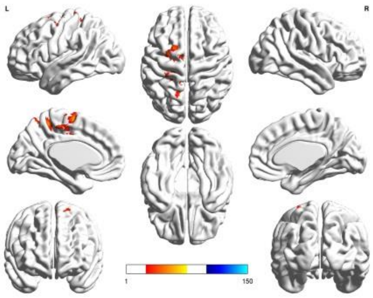

4C. Wernicke’s area connectivity

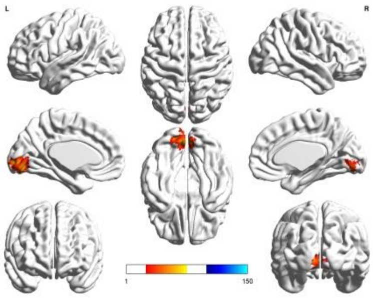

Children’s core language scores (CL) were positively correlated with connectivity between language networks and sensory networks across subjects, and there was no significant group differences in these relations; (A) CL was positively correlated with connectivity between Broca’s area and motor cortices (marked with red-yellow) (b) CL was positively correlated with connectivity between Broca’s area homologue and motor cortices (marked with red-yellow) (c) CL was positively correlated with connectivity between Wernicke’s area and visual cortices (marked with red-yellow), but there were no brain regions which showed correlation between CL and Wernicke’s area homologue connectivity.
